# Perilla Leaf Extract (PLE) Attenuates COPD Airway Inflammation *via* the TLR4/Syk/PKC/NF-κB Pathway *In Vivo* and *In Vitro*

**DOI:** 10.3389/fphar.2021.763624

**Published:** 2022-01-04

**Authors:** Jiqiao Yuan, Xuyu Li, Nan Fang, Ping Li, Ziqian Zhang, Mingbao Lin, Qi Hou

**Affiliations:** Institute of Materia Medica, Chinese Academy of Medical Sciences and Peking Union Medical College, Beijing, China

**Keywords:** PLE, COPD, airway inflammation, TLR4, Syk, PKC, NF-κB p65

## Abstract

Chronic obstructive pulmonary disease (COPD) is a complex and heterogeneous disease characterized by persistent airflow limitation but still lacking effective treatments. *Perilla frutescens (L.)* Britt.*,* an important traditional medicinal plant with excellent antioxidant and anti-inflammatory properties, is widely used for the treatment of respiratory disease in China. However, its protective activity and mechanism against COPD airway inflammation have not been fully studied. Here, the anti-inflammatory effects of the PLE were investigated, and its underlying mechanisms were then elucidated. The presented results suggested a notable effect of the PLE on airway inflammation of COPD, by significantly ameliorating inflammatory cell infiltration in lung tissue, lessening leukocytes (lymphocytes, neutrophils, and macrophages) and inflammatory mediators (interleukin 4 (IL-4), IL-6, IL-17A, interferon γ (IFN-γ), and tumor necrosis factor α (TNF-α)) in the bronchoalveolar lavage fluid (BALF) of cigarette smoke (CS)/lipopolysaccharide (LPS)-induced COPD mice *in vivo* and inhibiting the production of inflammatory factors (nitric oxide (NO), IL-6, and TNF-α) and intracellular reactive oxygen species (ROS) in LPS-stimulated RAW264.7 cells *in vitro*. For further extent, PLE treatment significantly suppressed the expression and phosphorylation of TLR4, Syk, PKC, and NF-κB p65 *in vivo* and their mRNA *in vitro.* Subsequently, by co-treating with their inhibitors *in vitro*, its potential mechanism *via* TLR4/Syk/PKC/NF-κB p65 signals was disclosed. In summary, the obtained results indicated a noteworthy effective activity of the PLE on COPD inflammation, and partly, the TLR4/Syk/PKC/NF-κB p65 axis might be the potential mechanism.

## Introduction

COPD, one of the most widespread chronic respiratory diseases with a prevalence of about 3.9% (Soriano et al., 2020) and the third leading cause of death (World Health Organization, 2019) worldwide, has not only impacted disastrously on mankind’s health but also caused heavy economic burden around the world (Soriano et al., 2017). Among the complex pathogenesis of COPD, chronic airway inflammation is thought to be a key driving factor in its pathological progression, which results in largely and gradually irreversible obstruction in the patient’s entire respiratory tract from the center to the periphery. Therefore, anti-inflammatory therapy has been an effective and key means in clinical treatment of COPD.

Currently, glucocorticoids are still recommended as initial maintenance treatment for COPD inflammation, alone or combined with bronchodilators. In severe COPD patients, long-acting β-agonist (LABA) or long-acting muscarinic antagonist (LAMA)/inhaled corticosteroid (ICS) combination treatment showed curative effects by controlling symptoms such as dyspnea and cough and preventing exacerbations. Although ICS treatment can reduce COPD airway inflammation, it is related with varieties of side effects, such as a higher risk of pneumonia (Rabe and Watz, 2017), and often developed corticosteroid resistance. Hence, the development of a new anti-inflammatory drug is still very urgent and imperative for COPD clinical treatment.

Traditional Chinese medicines (TCMs) have been reported to exhibit significant therapeutic effects on COPD by alleviating the symptoms, improving lung function, and quantity of life (Haifeng et al., 2015). *Perilla frutescens (L.)* Britt., a traditional Chinese herbal medicine, has been widely used in treating colds, coughs, nausea, vomiting, abdominal pain, constipation, asthma, and food poisoning in clinical settings in East Asia (China Pharmacopoeia Committee, 2015). Recently, much attention has been devoted to the anti-inflammatory function of its extracts on asthma (Yang H. et al., 2020; Yang et al., 2021). But its underlying effects and mechanisms on COPD have not been elucidated yet. Although asthma and COPD are both characterized by varying degrees of chronic airway inflammation and obstruction (Barnes, 2017), with considerable overlap in pathology and function (Woodruff et al., 2017), there are still many differences in their inflammation mechanisms. Therefore, our herein purpose is to investigate the activities of the PLE on COPD inflammation and to examine its possible mechanisms.

In stable COPD, Toll-like receptor 4 (TLR4), one of the major forms of innate immune sensors, was shown to associate with bronchial inflammation and bacterial load (Di Stefano et al., 2017). Spleen tyrosine kinase (Syk), a 72-kDa intracellular non-receptor tyrosine kinase, has emerged as a crucial effector molecule in TLR4 activation (Ma et al., 2020). Syk recruits through its tandem SH2 domains binding to dual-phosphorylated immunoreceptor tyrosine-based activation motifs (ITAMs) or ITAM-like sequences in C-type lectins, which then triggers kinase activation, leading to inflammatory responses *via* activating multiple downstream signaling, including protein kinase C (PKC) and nuclear factor-κB (NF-κB) (Mócsai et al., 2010). In light of these, the TLR4/Syk pathway is likely to contribute to the inflammatory progression of COPD.

In the present work, with the *in vivo* model of LPS/CS-stimulated COPD mice and the *in vitro* model of LPS-stimulated RAW264.7 macrophages, we assessed the activities of the PLE on COPD airway inflammation and then investigated its underlying anti-inflammatory mechanisms *via* the TLR4/Syk/PKC/NF-κB p65 pathway.

## Materials and Methods

### Extraction of the PLE

The PLE was from the same preparation (same batch number 20170622) of our previous research (Yang H. et al., 2020); the standardization of the PLE was also the same as before. Briefly, the dried *Perilla frutescens (L.)* Britt. leaves were ground into 40 meshes, then refluxed, and extracted twice by 5-fold (w/v) 70% alcohol, for 1.5 h per time. The combined extraction liquid was filtered and concentrated in a rotary vacuum evaporator, then ultrasonically mixed with water 3 times (w/v) to make a suspension and extracted twice by ethyl acetate. After the ethyl acetate in the water layer was volatilized, anhydrous ethanol was slowly added to the water layer and the mixture was stirred rapidly until the alcohol content was 80%, and the water layer was precipitated at room temperature for 24 h. Finally, the suspension was filtered to obtain the precipitant and supernatant. The precipitant was washed with 80% ethanol, and the washing solution was combined with the supernatant; then, the mixture was concentrated and dried to produce the PLE.

### Animals

Wild-type male Balb/c mice (6–8 weeks old, 20–22 g, Experimental Animal Center, Vital River Laboratory Animal Technology Co., Ltd, Beijing, China) were housed in standard environment-controlled conditions with chow diet and water provided *ad libitum*. The animal experiments were approved by the Animal Care & Welfare Committee of Institute of Materia Medica, Chinese Academy of Medical Sciences & Peking Union Medical College (Beijing, China) and conformed to internationally accepted ethical standards.

### LPS/CS-Induced COPD Airway Inflammation and Treatment

**FIGURE 1:**
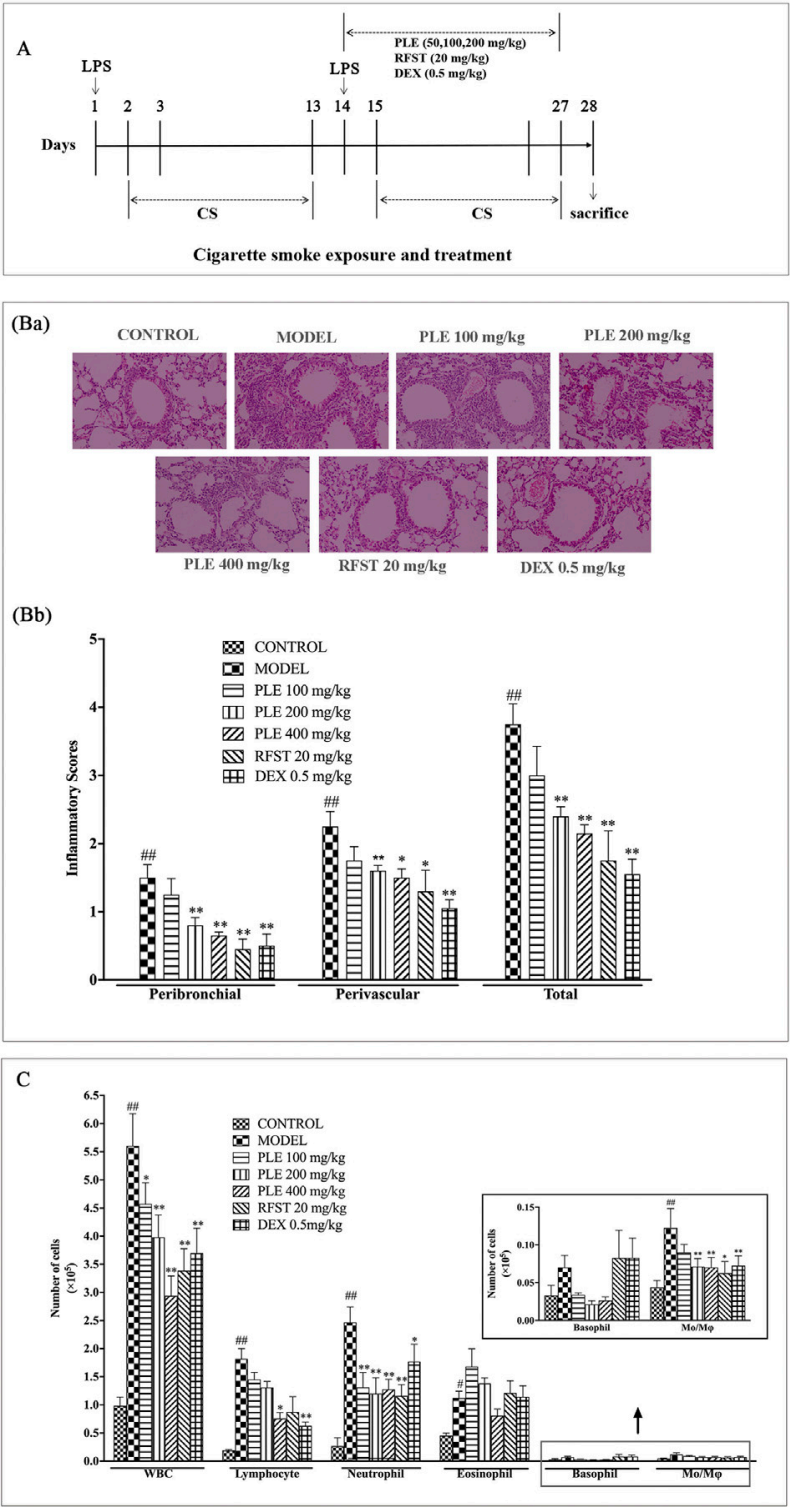
Effects of the PLE on lung inflammatory cell infiltration in COPD mice. **(A)** Timeline of the experimental protocol. **(Ba)** Typical histological images of H&E-stained lung tissues from LPS/CS-induced COPD mice (magnification ×100). **(Bb)** The inflammatory scores of H&E-stained lung tissues (5 independent slices from each mice, n = 3). **(C)** Total and differential leukocyte counts in the BALF (n = 8). Data were shown as mean ± SEM; ^#^ *p* < 0.05 and ^##^ *p* < 0.01 compared with the CONTROL group; *p < 0.05 and **p < 0.01 compared with the MODEL group.

LPS/CS-induced COPD airway inflammation of mice was induced, as described previously (Fan et al., 2019). Briefly, mice were divided randomly into seven groups: the control group, model group, PLE (100, 200, and 400 mg/kg) group, dexamethasone (DEX, 0.5 mg/kg) group, and roflumilast (RFST, 20 mg/kg) group. Except for the control group, mice were intratracheally instilled with LPS (40 µg in 50 µl phosphate buffer saline, PBS) on day 1 and day 14 and from day 2–27 (except on day 14), challenged with CS daily for 1 h in a microenvironment system for small animals (MBL-1; Institute of Materia Medica, Chinese Academy of Medical Sciences, Beijing, China). Animals were orally administrated daily from day 14–27, 1 h before air or CS exposure. 24 h after the last treatment, the mice were anesthetized and sacrificed. The timeline of the experiment was shown in Figure 1A.

### BALF Collection and Analysis

Twenty-four hours after the last administration, mice were anesthetized. Briefly, the BALF was collected by triple intratracheal instillation with 0.7 ml PBS buffer and centrifuged at 1,500 rpm for 10 min at 4°C to separate cells from the supernatant, and the enumeration for total white cells, neutrophils, monocytes, eosinophils, and lymphocytes was performed *via* a hemocytometer (Mindray BC-5000 vet, Shenzhen, China). The supernatants were removed and stored at −80°C for IL-6, IL-4, TNF-α, IFN-γ, and IL-17A detection, using commercial ELISA kits (R&D Systems) according to the manufacturer’s protocol with optical density (OD) values measured at 450 and 570 nm using a microplate reader (PowerWave XS2, BioTek, United States).

### Histological Examination

Lung tissue was harvested and fixed in 10% (v/v) formalin solution, then embedded in paraffin, sectioned at 4-μm thickness, applied to glass slides, and stained with hematoxylin and eosin (H&E) solution. Afterward, they were examined and photographed under a light microscope (100×). A four-point scale was used, as described previously (Yang H. et al., 2020). Generally, from each section, five blood vessels with a diameter of 100–200 μm or bronchi with a diameter of 150–300 μm were selected for examination. Histological examination was performed in a single-blind fashion.

### Cell Culture and Stimulation

RAW264.7 murine macrophage cells were cultured in Dulbecco’s modified Eagle medium supplemented with 10% (v/v) FBS, 100 U/mL penicillin, and 100 μg/ml streptomycin in a 37°C incubator with 5% CO_2_. 2 × 10^5^, 8 × 10^4^, or 2 × 10^4^ cells/well were seeded into 48-well plates for nitric oxide (NO), IL-6, or TNF-α determination, respectively. The cells were pretreated with the PLE (50, 100, 200 μg/ml), DEX (1 μM), TLR4 inhibitor (TAK242, 5 nM), Syk inhibitor (BAY61-3,606, 10 μM), PKC inhibitor (Rottlerin, 2.5 μM), and NF-κB inhibitor (BAY11-7,082, 5 μM) for 1 h and afterward, stimulated with LPS at 20 ng/ml for NO production, 5 ng/ml for IL-6 production, and 2.5 ng/ml for TNF-α production for 24 h. The culture supernatants were collected and used for detection.

### Cell Proliferation Assay

RAW264.7 cells were seeded at 2 × 10^5^/well in 96-well plates and were allowed to attach for 4 h before treatment with the PLE (50, 100, 200, 400, 800, 1,600, and 3,200 μg/ml) at 37°C for 24 h. Then, MTT (250 μg/ml) was added and incubated for 2 h. The OD values were measured at 570 nm using a microplate reader (Power Wave XS2, BioTek, Virginia, United States).

### Measurement of Intracellular ROS

The RAW264.7 cells, cultured in 24-well plates at 4 × 10^5^ per well, were used for intracellular ROS detection. The cells were pretreated with the PLE (50, 100, and 200 μg/ml) and DEX (1 μM) for 1 h and then stimulated with LPS (1 μg/ml) for 24 h. The cells were then incubated with 10 μM (final concentration) of the oxidant-sensing probe 2′,7′-dichlorodihydrofluorescein diacetate (DCFH-DA, Invitrogen, United States) at 37°C for 30 min in the dark. After cold PBS washing, their DCF fluorescence distribution were detected by using a flow cytometer (ACEA NovoCyte 2060R, China) and analyzed by FlowJo software (TreeStar Inc, Ashland, OR, United States). Images of DCF fluorescences in cells were captured by using an inverted research microscope (Nikon Eclipse Ti2, Tokyo, Japan) and analyzed by ImageJ software (National Institutes of Health, Maryland United States).

### Western Blotting Analysis

Mice lung tissues were lysed with RIPA buffer (Applygen, Beijing, China). The supernatants were collected and quantified with a BCA assay kit (Solarbio, Beijing, China) Then, protein extracts from tissues were separated on a 10% sodium dodecyl sulfate–polyacrylamide gel and transferred onto polyvinylidene difluoride membranes (Millipore, Bedford, MA, United States). After being blocked with 5% nonfat milk, the membranes were probed with specific primary antibodies against β-actin, TLR4, Syk, phospho-Syk (p-Syk) (Y525/526), PKC, p-PKC, NF-κB p65, and p-NF-κB p65 (Ser536) (all from Cell Signal Technology, United States) separately overnight at 4°C and incubated with horseradish peroxidase–conjugated anti-rabbit secondary antibody (Thermo Fisher, United States) for 2 h. The labeled protein bands were visualized using an enhanced chemiluminescent (ECL) substrate kit (YEASEN Biotech, Shanghai, China), and digital images were captured by a chemical imaging system (Clinx Science Instruments Co., Ltd, China). The density of each band was quantified by ImageJ.

### Quantitative Reverse Transcription–Polymerase Chain Reaction

By using Trizol (Transgen, Beijing, China), total RNA was isolated from RAW264.7 cells, which were pretreated with the PLE (50, 100, 200 μg/ml), DEX (1 μM), TLR4 inhibitor (TAK242, 5 nM), Syk inhibitor (BAY61-3,606, 10 μM), PKC inhibitor (Rottlerin, 2.5 μM), and NF-κB inhibitor (BAY11-7,082, 5 μM) for 1 h, respectively, and then stimulated with LPS (1 μg/ml) for 24 h. Random mixture primers and reverse transcriptase were used to synthesize the first-strand cDNA. Commercial primers were synthesized by XBiotech (Actb: DMM404349, Tlr4: DMM286110, Prkcd: DMM575032, Syk: DMM519482, Rela: DMM880760). qRT-PCR was performed in a real-time PCR machine (MYGO PRO, IT-IS, Ireland), following the instructions of the top green quantitative PCR SuperMix (Transgen, Beijing, China). A 2^−ΔΔCt^ method was employed to analyze the relative gene expressions by comparing with the internal control gene (β-actin).

### Statistical Analysis

Values were presented as the mean ± standard error of mean (SEM). Statistical analysis was performed by the Graphpad Prism 7.0 (GraphPad, La Jolla, CA) software and analysis of one-way ANOVA. Differences were considered statistically significant if *p* < 0.05.

## Results

### PLE Inhibited the Infiltration of Inflammatory Cells in LPS/CS-Induced COPD Mice

**FIGURE 2:**
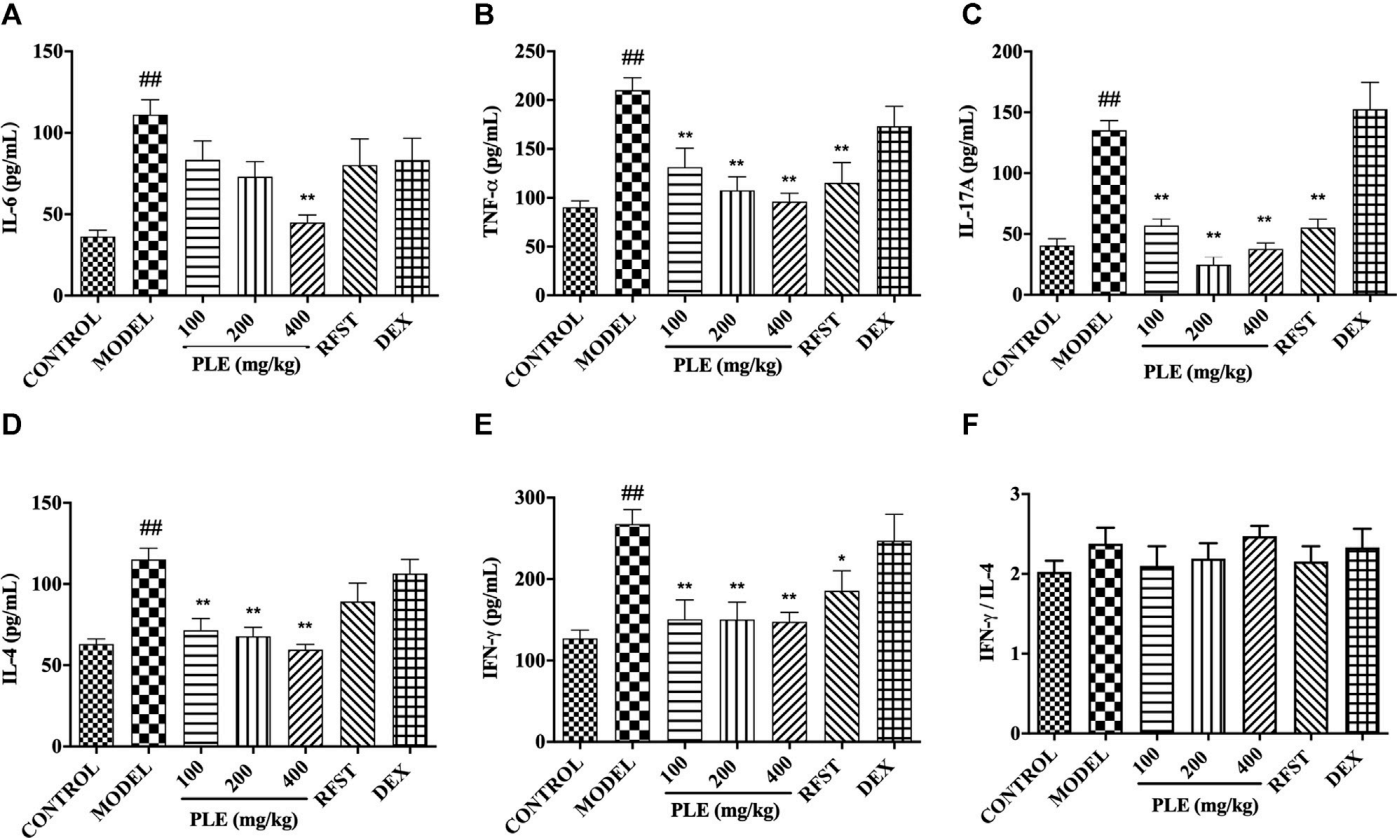
Effects of the PLE on the levels of inflammatory cytokines IL-6, **(A)** TNF-α, **(B)** IL-17A, **(C)** IL-4, and **(D)** IFN-γ **(E)**, and the ratio of IFN-γ/IL-4 **(F)** in the BALF. Data were shown as mean ± SEM, n = 8; ^##^ *p* < 0.01 compared with the CONTROL group; *p < 0.05 and **p < 0.01 compared with the MODEL group.

H&E staining and an inflammatory score were used to evaluate the inflammatory changes in the lung tissue of LPS/CS-induced COPD mice (Figure 2B). Extensive infiltration of inflammatory cells into peribronchial and perivascular regions was observed in COPD mice with significantly increased inflammatory scores, while, they were substantially ameliorated with PLE treatment (200 and 400 mg/kg, *p* < 0.01) and with RFST (20 mg/kg, *p* < 0.05 or *p* < 0.01) and DEX (0.5 mg/kg, *p* < 0.01) treatment.

Furthermore, as shown in Figure 1C, cell enumeration in the BALF also presented that PLE treatment and positive control RFST and DEX treatment markedly decreased the number of total white blood cells, lymphocytes, neutrophils, and macrophages (*p* < 0.01) dramatically, which were increased in COPD mice.

### PLE Inhibited the Production of Cytokines in the BALF From COPD Mice

The levels of inflammatory cytokines in the BALF were measured by an enzyme-linked immunosorbent assay. As shown in Figure 2, in agreement with the histological inflammatory changes, a memorably higher level of IL-6 (Figure 2A), TNF-α (Figure 2B), IL-17A (Figure 2C), IL-4 (Figure 2D), and IFN-γ (Figure 2E) was observed in COPD mice (*p* < 0.01), while they were significantly decreased with PLE treatment (*p* < 0.05 or 0.01), showing a dose-dependent manner in the decrease of IL-6, TNF-α, and IL-4. The ratio of IFN-γ/IL-4 was increased slightly but with no significance in COPD mice when compared with the control group. As to positive control, significant inhibitions were shown in TNF-α (*p* < 0.01), IFN-γ (*p* < 0.05), and IL-17A (*p* < 0.01) with RFST treatment, but no significance was observed with DEX treatment, the exact reason of which still needs to be explored. These results suggested that the PLE was imperative for ameliorating inflammation in LPS/CS-induced COPD mice and exhibited a better outcome in decreasing the levels of inflammatory factors with PLE treatment than RFST and DEX treatment.

### PLE Inhibited the Inflammatory Responses in LPS-Stimulated RAW264.7 Macrophages *In Vitro*

**FIGURE 3:**
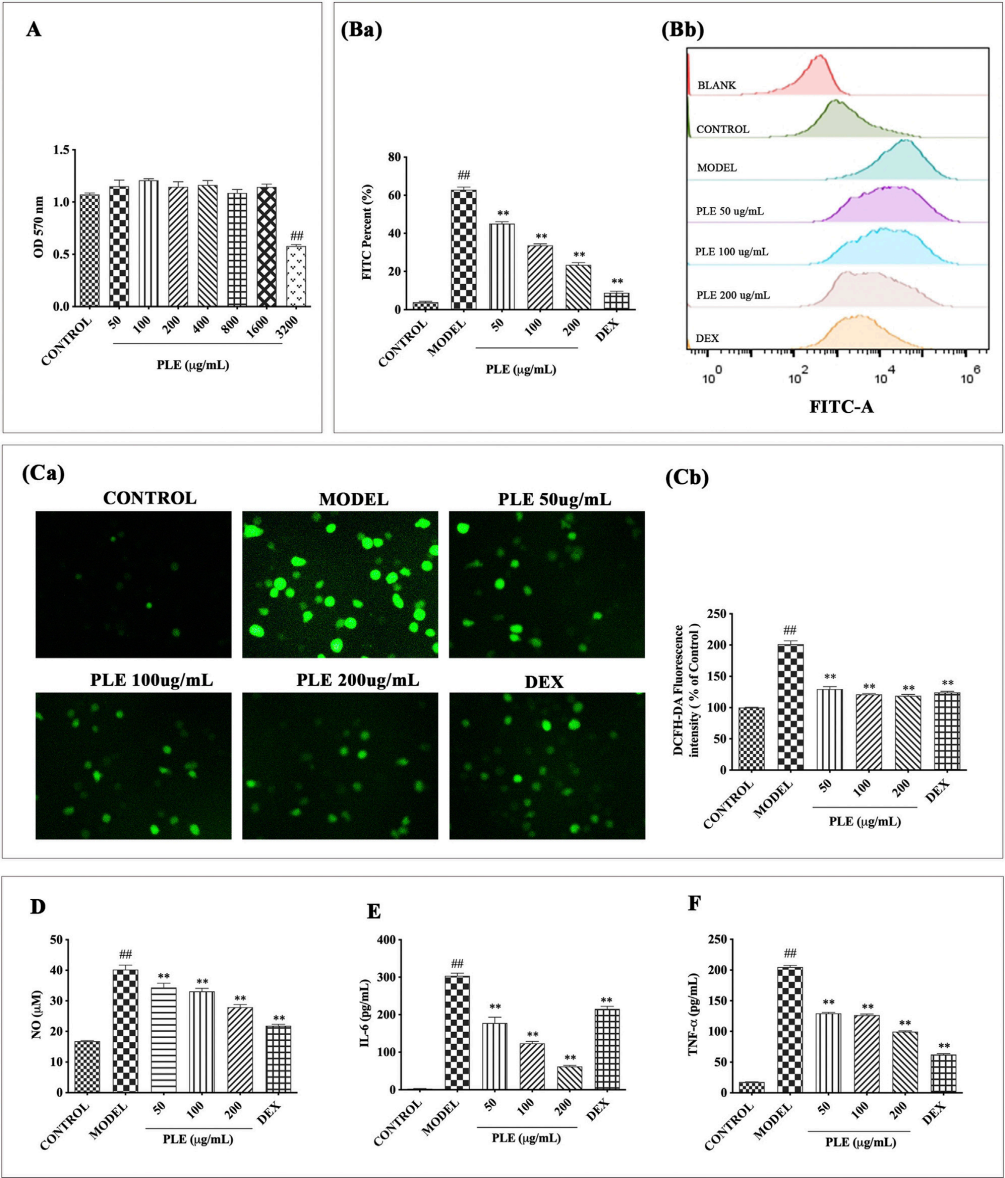
Effects of the PLE on inflammatory responses in LPS-stimulated RAW264.7 macrophages. **(A)** Cell proliferation was measured by MTT assay. **(Ba)** Intracellular ROS was determined by flow cytometry and **(Bb)** its DCFH-DA fluorescence shift was calculated by FlowJo software. **(Ca)** Representative images (magnification ×200) of cells DCF fluorescence visualized and **(Cb)** their intensity was analyzed by ImageJ software. Levels of NO **(D)** in cell culture supernatants were detected by Griess assay, and IL-6 **(E)** and TNF-α **(F)** were detected by ELISA assays. Experiments were carried out on 3 separate experiments. Data were shown as mean ± SEM. ^#^ *p* < 0.05 and ^##^ *p* < 0.01 compared with the CONTROL group; *p < 0.05 and **p < 0.01 compared with the MODEL group.

*In vitro* LPS-stimulated RAW264.7 cells were used to assess the anti-inflammatory effects of the PLE. As shown in Figure 3A, no obvious cytotoxicity was observed in PLE concentrations less than 1,600 μg/ml *in vitro*; therefore, 50, 100, and 200 ug/mL concentrations of the PLE were chosen for experiments *in vitro*. As shown in Figure 3B, PLE treatment significantly inhibited LPS-induced RAW264.7 cell intracellular ROS production (*p* < 0.01) manifested both in fluorescent cell counts and their representative fluorescent shift by flow cytometry detection, which was further confirmed by using an inverted research microscope observation (Figure 3C). As shown in Figures 3D–F, PLE treatment can significantly attenuate the secretion of NO (*p* < 0.01), IL-6 (*p* < 0.01), and TNF-α (*p* < 0.01) in LPS-stimulated RAW264.7 macrophages in a dose-dependent manner. By these, it is suggested that the PLE possessed an antioxidant and anti-inflammation potential *in vitro.*

### PLE Inhibited the Expressions of TLR4, Syk, p-Syk, PKC, p-PKC, NF-κB p65, and p-NF-κB p65 in Lung Tissues of COPD Mice

**FIGURE 4:**
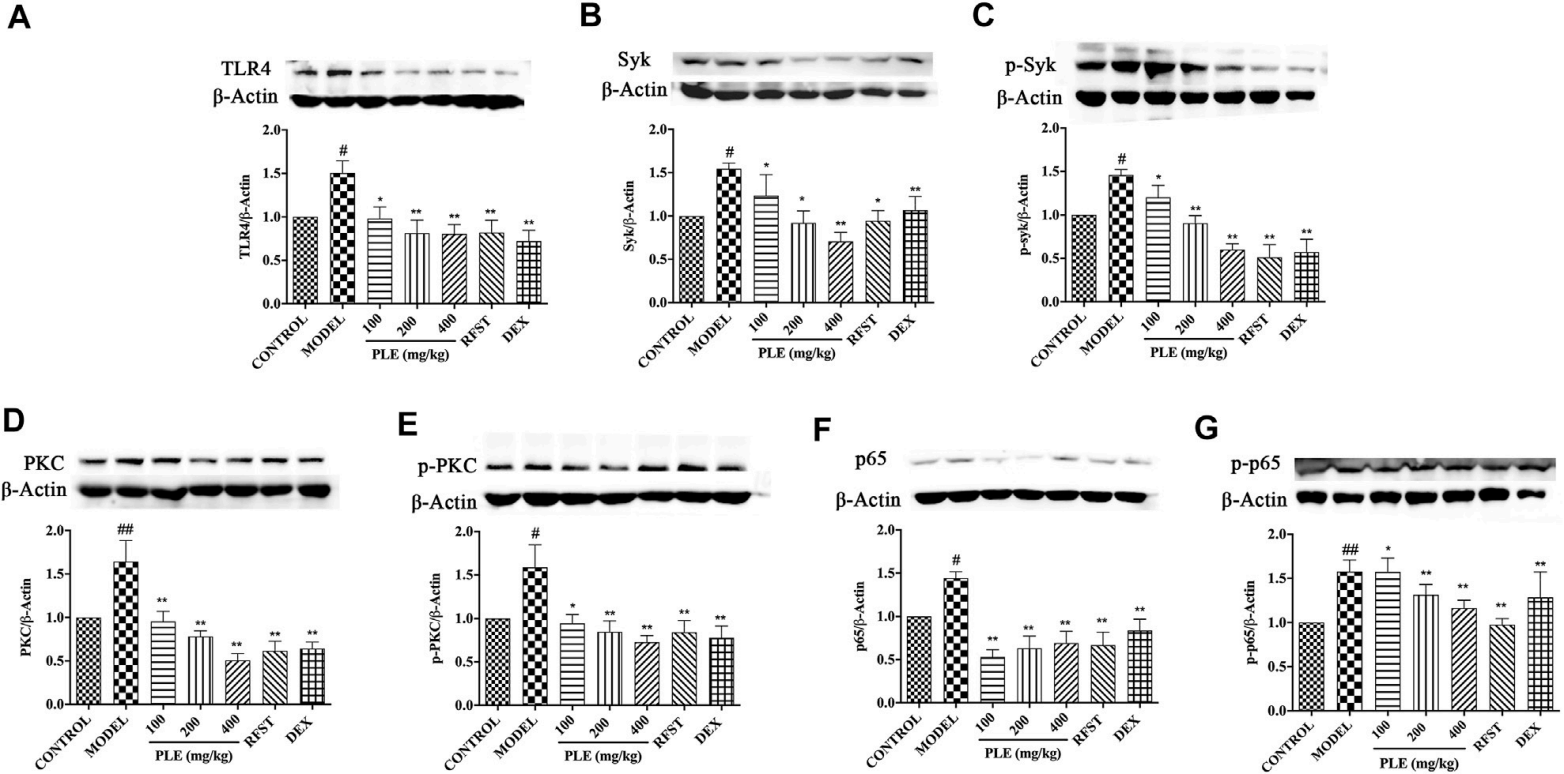
Effects of the PLE on the protein expressions of TLR4 **(A)**, Syk **(B)**, p-Syk **(C)**, PKC **(D)**, p-PKC **(E)**, NF-κB p65 **(F)**, and p-NF-κB p65 **(G)** in lung tissues of COPD mice (n = 5). Data were shown as mean ± SEM; ^#^ *p* < 0.05 and ^##^ *p* < 0.01 compared with the CONTROL group; *p < 0.05 and **p < 0.01 compared with the MODEL group.

*In vivo* significant increases of TLR4 (Figure 4A), Syk (Figure 4B), p-Syk (Figure 4C), PKC (Figure 4D), p-PKC (Figure 4E), NF-κB p65 (Figure 4F), and p-NF-κB p65 (Figure 4G) expressions were observed in lung tissues of LPS/CS-induced COPD mice (*p* < 0.05 or <0.01), while all of these increases were notably suppressed with PLE treatment (*p* < 0.05 or <0.01), acting dose dependently except that of the NF-κB p65 expression. These results indicated that the anti-inflammatory effects of the PLE on COPD might associate with inhibition of TLR4/Syk-related signaling pathways.

### PLE Decreased the Expression of TLR4, Syk, PKC, and NF-κB p65 mRNA in LPS-Stimulated RAW264.7 Cells *in vitro*

**FIGURE 5:**
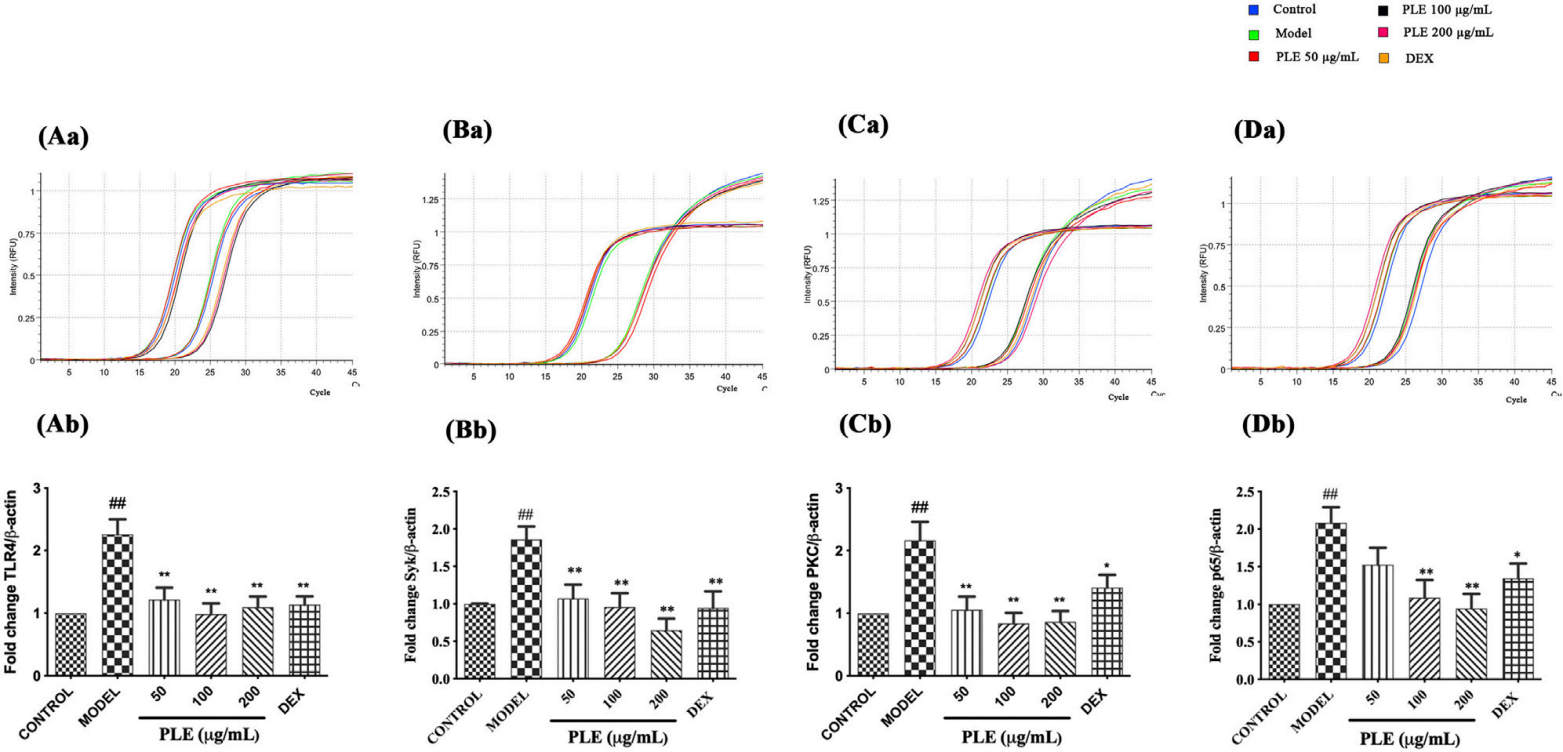
Effects of the PLE on the expression of TLR4 **(A)**, Syk **(B)**, PKC **(C)**, and NF-κB p65 **(D)** mRNA in LPS-stimulated RAW264.7 macrophages detected by qRT-PCR (n = 3). Representative images of the amplification curve of primer DMM286110 (Tlr4) **(Aa)**, DMM519482 (Syk) **(Ba)**, DMM575032 (Prkcd) **(Ca)**, and DMM880760 (Rela) **(Da)** as shown above. Data were shown as mean ± SEM; ^##^ *p* < 0.01 compared with the CONTROL group; *p < 0.05 and **p < 0.01 compared with the MODEL group.

In line with the *in vivo* expression of TLR4, Syk, PKC, and NF-κB p65, *in vitro* PLE treatment notably decreased the mRNA expression of TLR4 (Figure 5A), Syk (Figure 5B), PKC (Figure 5C), and NF-κB p65 (Figure 5D) which appeared to be as effective as DEX treatment (*p* < 0.05 or <0.01), suggesting its *in vitro* anti-inflammatory effect which is also associated with TLR4/Syk-related signaling.

### PLE Anti-Inflammation Effect *In Vitro* is Associated With the Inhibition of TLR4/Syk Signaling Pathway

**FIGURE 6:**
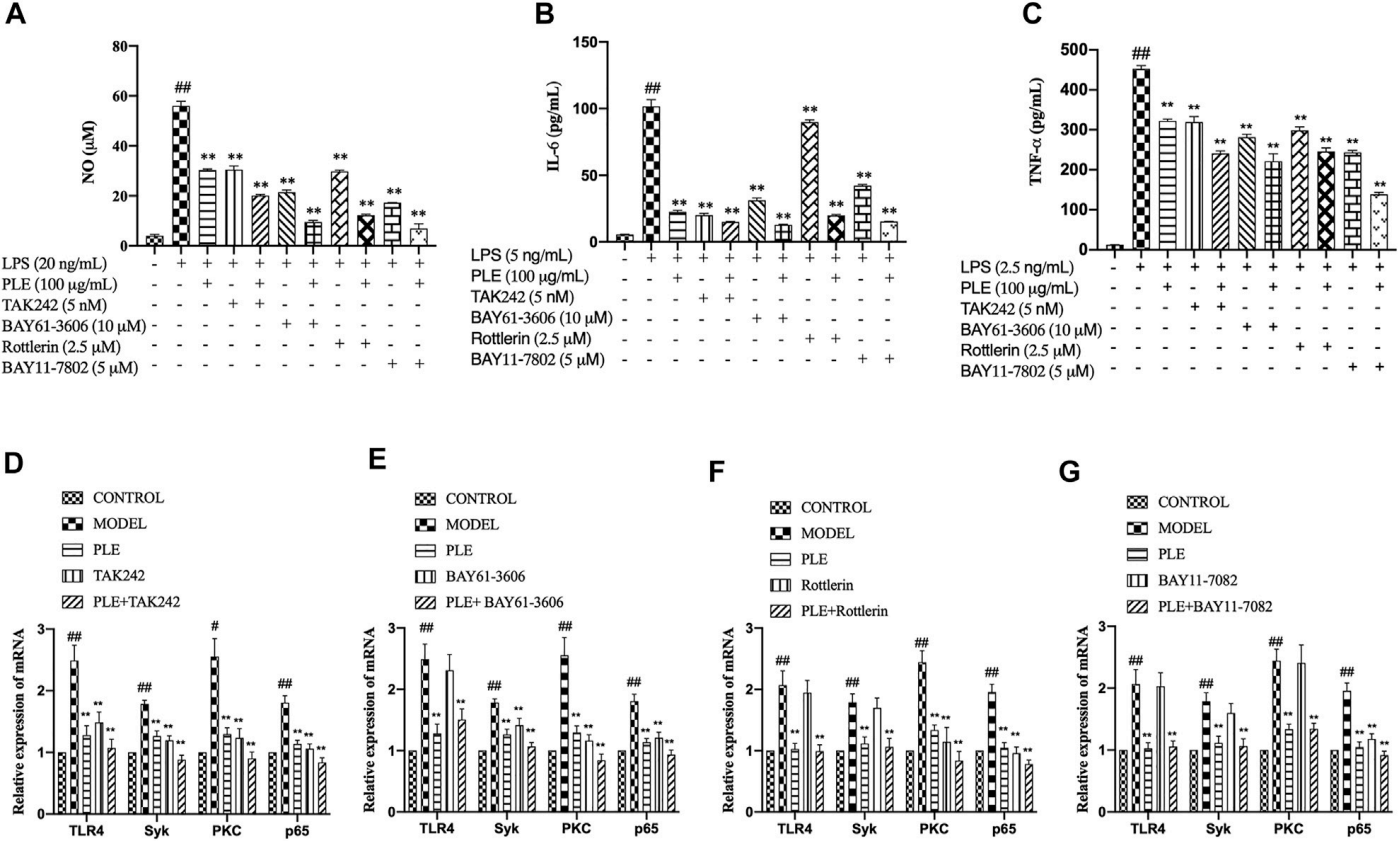
Verification that TLR4/Syk/PKC/NF-κB p65 were associated with PLE anti-inflammatory activity *in vitro*. Levels of NO **(A)** were detected by Griess assay, and those of IL-6 **(B)** and TNF-α **(C)** by ELISA assay. The mRNA expression of TLR4 **(D)**, Syk **(E)**, PKC **(F)**, and NF-κB p65 **(G)** were detected by qRT-PCR (n = 3). Data were shown as mean ± SEM. ^#^ *p* < 0.05 and ^##^ *p* < 0.01 compared with the CONTROL group; *p < 0.05 and **p < 0.01 compared with the MODEL group.

To further verify whether the PLE attenuated COPD airway inflammation *via* inhibition of the TLR4/Syk pathway, inhibitors of TLR4 (TAK242, 5 nM), Syk (BAY61-3,606, 10 μM), PKC (Rottlerin, 2.5 μM), and NF-κB p65 (BAY11-7,082, 5 μM) were employed to confirmatory study *in vitro*. Compared to individual treatment of PLE, TAK242, BAY61-3,606, Rottlerin, and BAY11-7,082 in LPS-stimulated RAW264.7 macrophages, the PLE co-treated with other inhibitors collaboratively suppressed the production of NO (Figure 6A), IL-6 (Figure 6B), and TNF-α (Figure 6C) (*p* < 0.01). Furthermore, by co-treating the PLE, TLR4 inhibitor (TAK242) obviously synergistically reduced TLR4, Syk, PKC, and NF-κB p65 mRNA expressions (Figure 6D); Syk inhibitor (BAY61-3,606) synergistically lessened the expression of Syk, PKC, and NF-κB p65 mRNA with no effect on TLR4 (Figure 6E); PKC inhibitor (Rottlerin) synergistically decreased PKC and NF-κB p65 mRNA levels with no effect on TLR4 and Syk (Figure 6F); and NF-κB inhibitor (BAY11-7,082) collaboratively inhibited the NF-κB p65 mRNA expression only (Figure 6G). Collectively, these results demonstrated that the PLE might mediate anti-COPD inflammatory response partly through TLR4/Syk/PKC/NF-κB p65 signals.

## Discussion

Inflammation has been described as the basic pathology of many human diseases. Chronic inflammation in COPD is a key driving factor in its pathological progression, mainly involving the infiltration of neutrophils, macrophages, lymphocytes, and other inflammatory cells into the airways and lung tissue (Hogg and Timens, 2009; Wang et al., 2018). For this, an effective and key means for COPD is anti-inflammatory therapy. But in clinical practice, a new anti-inflammatory drug is still very urgent, for many disadvantages and varieties of side effects existing in the current ICS treatment. Therefore, in the present study, by using LPS/CS-induced COPD mice *in vivo* and LPS-stimulated RAW264.7 macrophages *in vitro*, we attempted to demonstrate the therapeutic activities of the PLE, an extract from traditional Chinese herbal medicine *Perilla frutescens (L.)* Britt. The results presented showed that the PLE significantly mitigated extensive infiltration of inflammatory cells into peribronchial and perivascular sites *in vivo*, and LPS induced macrophages inflammatory responses *in vitro*, indicating a potential therapeutic capability for COPD.

In COPD airway inflammation, inflammatory cytokines play prominent roles, while important, which might be notably inhibited with PLE treatment. The increase or imbalance of the IL-6 expression is strongly implicated in the development of COPD, associated with progressive airflow obstruction and emphysema (Bradford et al., 2017). TNF-α induces an accumulation of inflammatory cells, provokes the generation of inflammatory mediators, and leads to oxidative and nitrosative stress, airway hyper-responsiveness, and tissue remodeling in COPD (Malaviya et al., 2017). IL-4 and IFN-γ were related with Th1/Th2 balance, IL-4 could act to promote lung-infiltrating monocytes differentiation into macrophages that produces MMP-12, resulting in the destruction of alveolar walls and emphysema development in COPD (Shibata et al., 2018), while IFN-γ synergistically enhanced TLR2 and TLR4 expressions and induced corticosteroid resistance in COPD patients (Southworth et al., 2012). Furthermore, Th17 cells also increase and secrete abundant IL-17A in the airways of COPD patients, which are involved in neutrophilic inflammation and corticosteroid resistance (Christenson et al., 2019). As to these inflammatory mediators, PLE treatment memorably decreased their production in the BALF of COPD mice *in vivo* and in LPS-stimulated RAW264.7 cells *in vitro*, further strongly demonstrating its potential therapeutic property on COPD inflammation. Our results were well consistent with the reported experimental observation. By CS and LPS exposure, cytokine IL-6, TNF-α, and protein NF-κB expression increased in the lungs of COPD mice, and airway inflammatory responses were effectively inhibited *via* the NF-κB pathway (Shin et al., 2018).

Furthermore, potential mechanisms of the PLE on COPD inflammation were discussed here. Previously, it was found that TLR4, Syk, PKC, and NF-κB play critical roles in inflammatory diseases (Slomiany and Slomiany, 2018; Yang H. et al., 2020; Ma et al., 2020), and the PLE and its several Syk affinitive components have been proved to act on Syk to relieve allergic airway inflammation (Yang H. et al., 2020) (Yang et al., 2021). TLR4 leads to initiation of Syk-, PKC-, and NF-κB p65-dependent signal cascades in COPD (Zhang et al., 2018; Fan et al., 2019; Yang L. et al., 2020). TLR4 is a receptor that mediates inflammatory responses to ligands, such as LPS from Gram-negative bacteria. And Syk, as a central immune modulator, may promote COPD airway inflammation through TLR4 and then trigger complex signals including PKC and NF-κB (Fan et al., 2019). PKC was involved in the pathogenesis of COPD and oxidative stress–associated pulmonary diseases. PKCβ and PKCδ inhibitors could act notably against mitochondria and cell oxidative damages in CSE-stimulated epithelial cells (Zhang et al., 2018). NF-κB, a critical trigger of inflammatory responses (Yi et al., 2014; Fan et al., 2019), triggers the excessive production of chemokines and cytokines. In the study, the results showed that the expression of TLR4, Syk, p-Syk, PKC, p-PKC, NF-κB p65, and p-NF-κB p65 in lung tissues of COPD mice were all notably suppressed with PLE treatment. Consistently, PLE treatment also significantly decreased TLR4, Syk, PKC, and NF-κB p65 mRNA expressions in LPS-stimulated RAW264.7 cells *in vitro*. The obtained results were in accordance with many other previous findings that COPD airway inflammation can be ameliorated by restraining the expression of TLR4 (Haw et al., 2018), Syk, PKC (Zhang et al., 2018), and p65 (Sun et al., 2019). These results indicated that the PLE exerts anti-inflammatory effects on COPD which might associate with its inhibition of TLR4/Syk/PKC/NF-κB p65 signaling pathways.

Macrophages are mononuclear leukocyte-derived key effector cells in orchestrating both the innate and adaptive immune responses, which increased in the airways, BALF, alveolar areas, and sputum of COPD patients (Akata and van Eeden, 2020). It has been reported that M1 pro-inflammatory macrophages are the predominant subpopulations in COPD (Chana et al., 2014), secreting many inflammatory mediators including TNF-α, IL-1β, IL-6, IL-12, IL-23, IL-27, CXCL9, CXCL10, CXCL11, CXCL16, CCL5, and ROS (Shapouri-Moghaddam et al., 2018). Therefore, LPS-stimulated RAW264.7 macrophages were used to further verify the possibility that TLR4/Syk/PKC/NF-κB p65 is the functional target of the PLE on COPD inflammation. By co-treatment of inhibitors of TLR4, Syk, PKC, and NF-κB p65 with the PLE respectively, an additive inhibition of inflammatory mediators, such as NO, IL-6, and TNF-α, was exhibited, indicating that TLR4, Syk, PKC, and NF-κB p65 can contribute to PLE treatment alleviating COPD airway inflammatory mediator generation. Furthermore, co-treating with the PLE, the TLR4 inhibitor (TAK242) synergistically reduced the TLR4, Syk, PKC, and NF-κB p65 mRNA expression; Syk inhibitor (BAY61-3,606) synergistically lessened the expression of Syk, PKC, and NF-κB p65 mRNA with no effect on TLR4; and PKC inhibitor (Rottlerin) synergistically decreased PKC and NF-κB p65 mRNA levels. But the relationship between Syk and PKC is still controversial. Some researchers demonstrated that PKC was able to activate the Syk signaling pathway in inflammation-induced macrophage phagocytosis of healthy self-cells (Bian et al., 2016), whereas others suggested that Syk seemed to be the upstream of PKC in the mechanism of neutrophil-mediated killing of *C. albicans* (Gazendam et al., 2014), and the NF-κB inhibitor (BAY11-7,082) only inhibited the expression of NF-κB p65 mRNA. It has also been shown that the PLE significantly diminished the expression of a pro-inflammatory cytokine IL-6 through inhibition of NF-κB activation to exert its anti-inflammatory activity *in vitro* (Liu et al., 2013). Taken together, the results suggested that the PLE exerted its anti-COPD inflammation potentially *via* TLR4/Syk/PKC/NF-κB p65 signals.

In conclusion, the presented study unveils the ameliorated effects of the PLE on COPD inflammation *in vivo* and *in vitro*, suggesting its potential therapeutic usages in COPD treatment and partly, through blocking the TLR4/Syk/PKC/NF-κB p65 signal pathway. But because of the limited time and experimental conditions, we mainly focused on NF-κB and its upstream TLR4/Syk/PKC-related signaling pathway in this preliminary research stage; some mechanisms were not explored. The pathogenesis of COPD is complicated and unclear and related to many important pathways, such as MAPK-related signaling pathways, ERK1/2, ERK5, p38, and JNK, which play prominent roles in the regulation of the innate and adaptive immune response (Liu et al., 2007; Pereira and Rodrigues, 2020; Wang et al., 2020). Consequently, we will dig into whether the MAPK signaling pathway is involved in COPD airway inflammation and the relationship between NF-κB and MAPK signaling pathways. In addition to the foregoing limitations, compounds in the PLE have the complexity and diversity, and the exact effective components and whether they have pharmacodynamic synergy also need defining.

## Data Availability

The original contributions presented in the study are included in the article/Supplementary Material, further inquiries can be directed to the corresponding authors.
